# Electrostatic Switch Function in the Mechanism of Protein Kinase A I**α** Activation: Results of the Molecular Dynamics Simulation

**DOI:** 10.1155/2017/5846073

**Published:** 2017-03-07

**Authors:** Olga N. Rogacheva, Boris F. Shchegolev, Elena A. Vershinina, Alexander A. Tokmakov, Vasiliy E. Stefanov

**Affiliations:** ^1^St. Petersburg State University, Universitetskaya nab. 7/9, St. Petersburg 199034, Russia; ^2^Pavlov Institute of Physiology, Russian Academy of Sciences, nab. Makarova 6, St. Petersburg 199034, Russia; ^3^Kyoto Sangyo University, Kamigamo-Motoyama, Kita-ku, Kyoto 603-8555, Japan

## Abstract

We used molecular dynamics to find the average path of the A-domain H → B conformational transition in protein kinase A I*α*. We obtained thirteen productive trajectories and processed them sequentially using factor and cross-correlation analyses. The conformational transition is presented as partly deterministic sequence of six events. Event B represents H → B transition of the phosphate binding cassette. Main participants of this event form electrostatic switch cAMP(O6)–A202(N-H)–G199(C=O). Through this switch, cAMP transmits information about its binding to hydrophobic switch L203–Y229 and thus triggers conformational transition of A-domain. Events C and D consist in N3A-motif displacement towards phosphate binding cassette and B/C-helix rotation. Event E involves an increase in interaction energy between Y229 and *β*-subdomain. Taken together, events B, E, and D correspond to the hinge movement towards *β*-barrel. Transition of B/C-helix turn (a.a. 229–234) from *α*-form to *π*-form accounts for event F. Event G implies that *π*-helical turn is replaced by kink. Emerging in the resulting conformation, electrostatic interaction R241–E200 facilitates kink formation. The obtained data on the mechanism of cAMP-dependent activation of PKA I*α* may contribute to new approaches to designing pharmaceuticals based on cAMP analogs.

## 1. Introduction

Cyclic nucleotides since their initial discovery have been attracting interest of researchers in physiology and biomedicine due to the ability of these compounds to act as secondary messengers. It was shown that the cAMP analogs such as Rp-cAMPS and its derivatives are effective protein kinase A I*α* inhibitors and therefore could be used for treatment of pathological states when the cAMP/PKA I*α* path is hyperactivated [1]. Optimization of anti-HIV pharmaceutical agents based on Rp-cAMPS is one of the challenging problems in practical medicine. It implies a deeper insight into the mechanism of protein kinase A I*α* activation. The detailed analysis of the enzyme interaction with cAMP at the molecular level including functioning of electrostatic and hydrophobic switches should be based on the available data on detected sequences and point mutations.

A-domain of protein kinase A (PKA) regulatory subunit RI*α* is an example of cAMP-binding domains, which are widely spread among different proteins and thoroughly studied in various methods. In spite of this, average paths of cAMP-induced transition from H-conformation (cAMP-free) to B-conformation (cAMP-bound) are not determined for any members of this domain family, presenting a major challenge for further research. In order to explain conformational changes of these domains (particularly, A-domain of RI*α*), two allosteric switches were proposed. The first one is the hydrophobic switch L203–Y229 that contains hinge region D227–I232 and links B/C-helix (Y229) directly to L203 of phosphate binding cassette (PBC) [2, 3]. The second one is the electrostatic relay R209–D170–R226 that links B/C-helix (R226) to PBC (R209) through the *β*2*β*3-loop (D170) [4, 5]. Both switches can assume two distinctive ‘‘on” and ‘‘off” positions so that switching between them causes the domain to undergo a transition between H-conformation and B-conformation. The hydrophobic switch model assumes that in H-conformation hinge movement is blocked; otherwise Y229 would clash into L203; cAMP binding reorients the leucine and produces a large movement of the hinge and region C-terminal to it (lid region) toward the ligand. According to the electrostatic relay model, cAMP interacts with R209 side chain and forces D170 to switch from R226 to R209. This process allows B/C-helix to sense cAMP binding. The first model is proposed and confirmed experimentally by Rehmann et al. who demonstrated that replacement of L203 with amino acids with bulky side chains abolishes transition to B-conformations, whereas replacement of Y229 with amino acids with small side chains has the opposite effect [2]. Unfortunately, the mechanism of cAMP-dependent activation of this switch remains unknown. In contrast, the electrostatic switch model explains how information about cAMP binding is transferred outside the cAMP-binding site. However, this model cannot account for the activation of PKA with mutational substitutions for R209 [6]. Some of these substitutions (R209I, R209T) have a minor influence on PKA activation, though they obviously uncouple the electrostatic relay. In accordance with these experimental findings, we recently demonstrated that mutational substitution R209I does not block the transition from H-conformation to B-conformation or affect the stability of the latter [7].

In order to elucidate the roles of both switches in the conformational transition of A-domain and to clarify the mechanism of this transition, we performed molecular dynamics (MD) and accelerated MD simulations followed by factor and cross-correlation analyses. These results constitute a first approximation and provide the foundation for further calculations of the minimum free energy path.

## 2. Experimental Procedures

The spatial structure of A-domain (a.a. 118–241) in H-conformation was taken from PDB entry 3PVB [8], and docking of the cAMP into ligand-binding site was performed as it was described previously [9]. In addition to the wild type domain with arginine residue in position 209, the domain carrying point mutation R209K was modeled. This mutation was introduced using NAMD 2.8 standard options [10]. The resulting wild type or mutant A-domains were subjected to MD calculations.

Simulations were performed with NAMD 2.8 software under the periodic boundary conditions and aqueous environment using the CHARMM27 force field and a time step of 2 fs [10–12]. The electrostatic component of interactions was taken into consideration by Particle Mesh Ewald algorithm [13]. According to the existing data, G169, being a part of the *β*2*β*3-loop, forms a CH–*π* interaction with the guanidinium group of R209, which maintains the peculiar conformation of the sought loop [14]. Preliminary calculations showed that the CHARMM27 force field has no parameters correctly describing this interaction; therefore, in spite of R209 presence, the *β*2*β*3-loop passes into another conformation during the first nanosecond of simulation. To avoid this unfavorable effect, we exposed the atoms of the main chain of the *β*2*β*3-loop to the forces that compel the loop to stay in the position corresponding to B-conformation (force constants were 4-5 kcal/mol per Å^2^). The same forces were applied to the atoms of K209 side chain in a structure with R209K substitution. The goal was to hold the side chain in position that is close to R209 position in wild type protein. These restrictions were applied in accordance with our previous findings, which show that lysine does not interfere with A-domain transition if its side chain is fixed at the same place as side chain of wild type arginine [7].

During the first 100 ps, systems were heated and then simulated under NVE ensemble with mean kinetic temperature close to 350 K. This stage of simulation lasted until PBC reached B-conformation but not longer than 10 ns. Other stages of transition were conducted under NVT ensemble at 300 K maintained with Langevin dynamics with damping coefficient *γ* of 5 ps^−1^. The second stage lasted until the hinge motion occurred. The third stage differed from the second one due to the use of accelerated MD [15, 16], where boost potential was applied to dihedral angles with threshold energy *E* of 800–1000 kcal/mol and acceleration factor *α* of 2 kcal/mol. Accelerated MD was used in order to improve transition of B/C-helix turn from *α*-form to *π*-form. This stage was omitted in some simulations because transition from *α*-form to *π*-form occurred spontaneously. The fourth stage was unaccelerated and lasted until the kink began to replace the *π*-helical turn. As this process was slowed down by transient salt bridge formation between C-helix (K240 or R239 residues) and N3A-motif (D140 residue), we introduced two mutations (K240A and R239A) and continued the simulation until complete kink was reached. Resulting structures were equilibrated for 10–15 nsec and compared with a known B-conformation available in PDB (entry 1NE6) [17]. In order to perform this comparison, we chose the following four parameters: root mean square distances (RMSD) between backbone atoms of received structures and B-conformation for PBC, N3A-motif, B/C-helix, and whole A-domain. Corresponding values amounted to 0.63 ± 0.01, 1.93 ± 0.11, 1.68 ± 0.11, and 1.20 ± 0.05 Å, respectively, for wild type domain and 0.62, 1.56, 1.27, and 0.98 Å for domain with the R209K substitution. The result confirms that in the course of simulation A-domain came into the B-conformation.

In total, we obtained thirteen transition paths, or trajectories, of A-domain from H-conformation to B-conformation (twelve for wild type protein and one for R209K mutant) with overall mean time of 40–45 ns (transition time was about 30 ns). For the specification of A-domain conformational transition, we chose twenty-six collective variables listed in Supplement. Time-dependent changes of these variables were expected to be sufficient to describe the conformational lability of the A-domain.

We started by cutting all trajectories into several parts in order to increase the accuracy of further analysis. To prevent the protein motion detection impairment caused by the cutting of trajectories, the ends of these parts were assigned to plateau segments in all collective variables graphs.

All trajectory parts were sequentially subjected to factor analysis (performing principal components method by using varimax rotation) and cross-correlation analysis on SPSS software [18]. Factor analysis was performed on time-dependent data, so factor coordinates that are projections of original 26-dimensional data on extracted factors also represent time functions. However, unlike the original collective variables, the last can be associated with collective slow protein motions that are the basis for protein conformational transition. We selected only factors that correspond to one-way motions and located these motions in time by cross-correlation analysis. This analysis was performed by pairwise comparison of factor coordinates' time behavior. Thus, for each path, we arranged and analyzed factors in order of happening of corresponding domain motions (Supplemental Tables 1–13 in Supplementary Material available online at https://doi.org/10.1155/2017/5846073).

We expected that different trajectories would be alike, though not identical. This prediction was confirmed by the comparison of factor structure between different trajectories (Supplemental Tables 1–13). We extracted common factors with similar values of factor loadings. We hypothesized that if absolute values of several loadings always reached their maximum within the same factor (we highlighted the cells with these loadings with the same color), corresponding changes of collective variables accounted for the discrete atomic event in the course of conformational transition. It should be noted that one factor can comprise more than one event, and common factors do not necessarily involve exactly the same set of events. We extracted six events that account for A-domain conformational transition and presented the transition as the sequence of these events.

Visualization of protein structures was performed with VMD software [19].

## 3. Results

The data for all trajectories are summarized in Supplemental Table 14. Visual analysis of this table gave us the most probable sequence of events: (1) B; (2) C1 and D1; (3) E; (4) D2[C2] and F; (5) G. This sequence can be treated as a first approximation for the minimum free energy path of A-domain cAMP-induced conformational transition. The sequence corresponds to the process of the conformational transition in time. Events listed under the same number can occur in any order, including the situation when any event occurs against the background of another one. By contrast, events described under different numbers proceed in a certain order: each of the events under higher number may occur simultaneously with the events of the lower number but not earlier. It should be noted that the only transition path of A-domain with R209K substitution does not differ from the wild type pathways more than these pathways differ among themselves. Thus, we confirmed our previous data that explain the role of residue at position 209 in PKA I*α* function [7].

**Figure 1:**
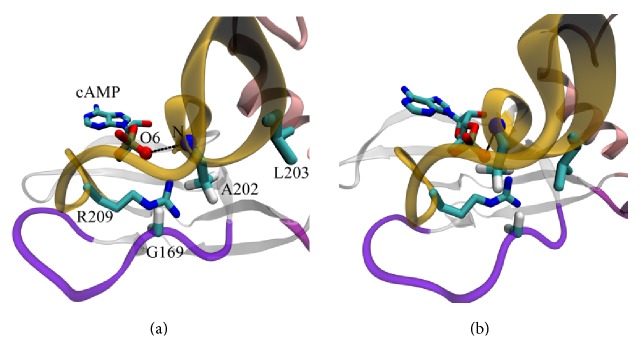
A-domain conformational transition, event B. (a) Before event B (H-conformation). (b) After event B. Colored beads show key atoms of the cAMP(O6)–A202(N-H)–G199(C=O) electrostatic relay: exocyclic oxygen O6 and amide nitrogen of A202 are shown with beads, with black line denoting distance between these atoms equal to 5.2 and 3.4 Ǻ in H-conformation and B-conformation, respectively. In (a), L203 side chain is directed away from the hydrophobic pocket formed by *β*2*β*3-loop. In (b), L203 is packed into the hydrophobic pocket. Here and elsewhere, colored ribbons show elements of the secondary structure: rose, N3A-motif; dark yellow, PBC; violet, *β*2*β*3-loop; purple, B/C- or B- and C-helices; separately highlighted are critical residues and cAMP.

Event B (Figure 1, Supplemental A-domain_transition.mov) represents the PBC transition from H-conformation to B-conformation and triggers the transition of the whole domain. It was discussed in detail previously [7, 9]. Briefly, the amide group of A202 residue moves toward the equatorial exocyclic oxygen of the cAMP (O6). This results in the disruption of G199(C=O)–A202(N-H) H-bond, subsequent transformation of PBC short helix from 3_10_ to *α*-form, and packing of the L203 side chain into the hydrophobic pocket formed by *β*2*β*3-loop. Main participants of this event (amide group A202, carbonyl group G199, and equatorial oxygen atom of the ligand) hereinafter are referred to as the electrostatic switch cAMP(O6)–A202(N-H)–G199(C=O). Through this switch, cAMP transmits information about its binding to the hydrophobic switch L203–Y229 and thus triggers the conformational transition of A-domain.

**Figure 2:**
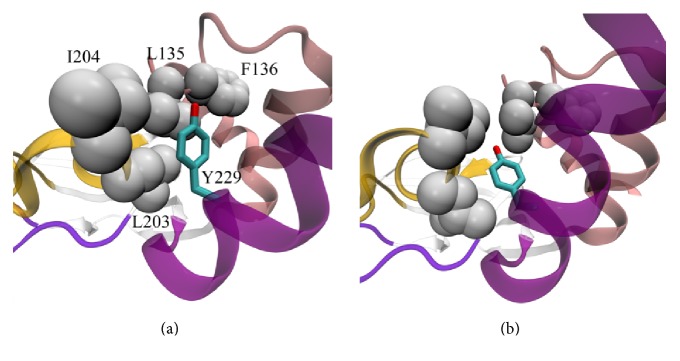
A-domain conformational transition, events C, D, and E. cAMP, though present in the binding site, is not shown in Figures 2–4. (a) Before events C, D, and E. (b) After events C, D, and E. Side chains of L203, I204, L135, and F136, which form hydrophobic cluster in H-conformation, are shown with grey spheres of van der Waals radii. In (a), L203 side chain is not packed into the pocket formed by *β*2*β*3-loop, and the hydrophobic cluster is still stable. In (b), the hydrophobic cluster is destroyed by the L203 packing into the hydrophobic pocket and N3A-motif displacement, B/C-helix is rotated, and Y229 aromatic ring is placed upon L203 side chain, approaching *β*-subdomain.

Event C (Figure 2, Supplemental A-domain_transition.mov) consists of N3A-motif displacement away from the PBC. It can be hypothesized that event C is triggered by L203 and I204 residues reposition that is the result of event B. In H-conformation, side chains of PBC residues L203 and I204 and side chains of N3A-motif residues L135 and F136 are tightly packed together and form a hydrophobic cluster as shown in Figure 2(a). In this cluster, the distance between С*α* atoms of L135 and I204 is approximately 8 Å. PBC helix transition leads to the displacement of L203 and I204 residues and the destabilization of the hydrophobic cluster. The probability of the cluster disruption increases, and once it happens N3A-motif begins to move away from PBC. In B-conformation, there is no interaction between PBC and N3A-motif, and the final distance between С*α* atoms of L135 and I204 reaches 15 Å. During this process, B/C-helix also loses its contacts with N3A-motif. However, further transition leads to the recovery of the interaction between B/C-helix and N3A-motif. It is worth pointing out that cAMP-binding domains of EPACs structures demonstrate no difference in N3A-motif position between H-conformation and B-conformation [20, 21]. In both cases, N3A-motif is displaced from the PBC, and its position is in good agreement with the structure of PKA I*α* A-domain in B-conformation [17]. This is why event C was overlooked in previous studies of hinge motion carried out on cAMP-binding domain of EPAC [2, 3].

Event D (Figure 2, Supplemental A-domain_transition.mov) involves a 45° rotation of B/C-helix in the plane of Figure 2(a). This event is initiated by L203 side chain packing into the hydrophobic pocket, the process previously described as part of event B. It was observed [2, 3] that in H-conformation side chain of L203 collides with the aromatic ring of B/C-helix residue Y229, effectively preventing the rotation of B/C-helix. After the side chain of L203 gets into the hydrophobic pocket, this hindrance is removed, and the helix performs a 45° rotation. This scheme was first proposed by Rehmann as a basis for his hinge model. Our simulations fully confirm this model and allow us to clarify the reasons behind the B/C-helix rotation. According to our findings, this rotation is driven at first by the preservation of Y229 and L203 interaction and then by the increase in interaction energy between Y229 and *β*-subdomain. Moreover, an essential prerequisite for B/C-helix rotation is the displacement of N3A-motif (event C). It is so because this motif in H-conformation and B-conformation and C-helices in B-conformation occupy the same segment of space in PKA cAMP-binding domains.

During the conformational transition, Y229 hydrophobic environment undergoes significant changes. Firstly, L203 side chain packs into the hydrophobic pocket (event B). The interaction between L203 and Y229, destabilized by this process, seems to be favorable and is restored completely when B/C-helix rotation angle reaches 20–30° (a part of event D). Secondly, N3A-motif starts to move away from PBC (a part of event C). This displacement reduces the contribution of L135 and F136 residues in Y229 hydrophobic environment. Contacts between these residues and Y229 begin to break as the distance between N3A-motif and PBC increases. However, as L135 and F136 move away, they no longer shield Y229 from the residues of *β*-subdomain (L203, I204, E200, and F172). Being in the vicinity of these residues as the result of events B and D and having access to them due to event C, Y229 acquires the ability to increase the energy of its interaction with *β*-subdomain. Event E constitutes the transformation of this ability into action. Structural change corresponding to event E can be described as a process where Y229 aromatic ring is placed upon L203 side chain, approaching *β*-subdomain (Figure 2(b), Supplemental A-domain_transition.mov). As a result of event E, interaction energy between Y229 and the residues of *β*-subdomain increases approximately twofold.

The diagram in Supplemental Table 14 demonstrates that events C and D are characterized by duration varying with simulation run and irregularity of ongoing processes. However, certain regularity in the behavior of these events still exists. Due to their duration, events C and D tend to occur in two stages labeled with marks “1” and “2.” Event E defines the boundary between these stages. Before event E, B/C-helix has to rotate to reach a 20–30° angle and the hydrophobic cluster linking N3A-motif and PBC has to be destabilized without a significant increase in the distance between L135 and I204 С*α* atoms. This stage together with event B enables event E. Further displacement of N3A-motif may occur before event E, but in our simulations it happened infrequently. Further rotation of B/C-helix is the consequence of event E and also depends on N3A-motif displacement, if N3A-motif has not reached the position characteristic of B-conformation prior to event E. As event C very likely affects the progress of event D, we denoted this dependence by D2[C2] and included it in the aforementioned sequence of events.

**Figure 3:**
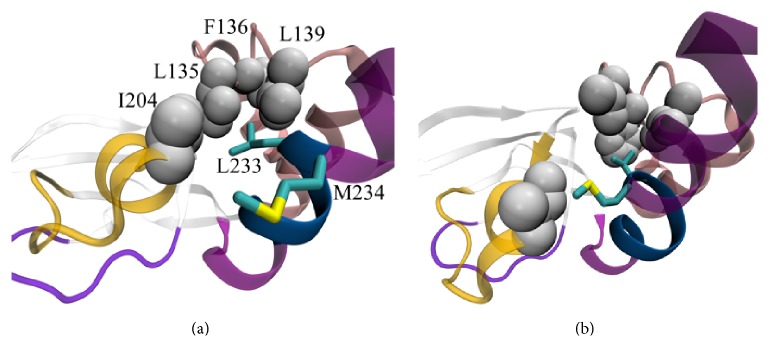
A-domain conformational transition, event F. (a) Before event F. (b) After event F. Side chains of I204, L135, F136, and L139 are shown with grey spheres of van der Waals radii. In (a), L233 and M234 do not form favorable hydrophobic contacts with other residues. In (b), L233 and M234 rotate to N3A-motif and PBC, respectively, and acquire a favorable hydrophobic environment. This rotation is accompanied by transition of B/C-helix turn (a.a. 229–234, marked with dark blue) to *π*-form, which exhibits a lower curvature degree as compared with *α*-form.

Event F (Figure 3, Supplemental A-domain_transition.mov) consists in B/C-helix turn (a.a. 229–234) transition from *α*-form to *π*-form and the simultaneous rotation of M234 side chain to the PBC (I204) and L233 side chain to the N3A-motif and *β*-subdomain (L135, F136, L139, and F172). It can be assumed that event F resulted from L233 and M234 residues' tendency to create a favorable hydrophobic environment and, therefore, increase the energy of interaction with nearby residues. In this case, the formation of the *π*-helical turn is forced by the need for a local decrease in helical curvature, which in turn is required to enable correct positions of L233 and M234 side chains.

**Figure 4:**
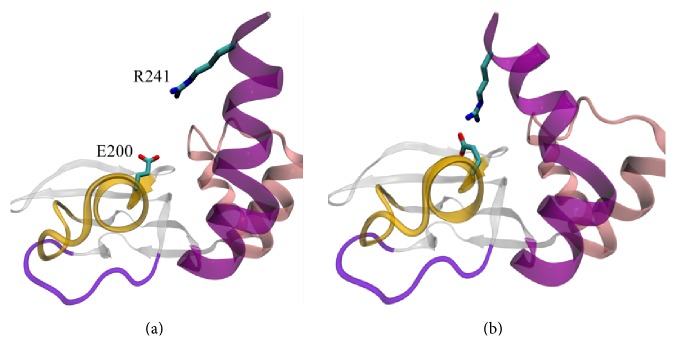
A-domain conformational transition, event G. (a) Before event G. (b) After event G (B-conformation). B/C-helix turn, which is in *π*-form following event F (a), is replaced by kink breaking up the B/C-helix into separate B-helix and C-helix (b). Formation of R241-E200 salt bridge (b) facilitates this process.

Event G (Figure 4, Supplemental A-domain_transition.mov) is the last in a chain of events that define the A-domain conformational transition. During event G, a kink replaces the *π*-helical turn, and two interactions (the first between R241 and E200 side chains and the second between L238 and I204 side chains) present in X-ray structure of B-conformation [17, 22] facilitate this process. Both interactions seem to favor the achieved angle between B-helix and C-helix. This guess can be confirmed by the recovery of single B/C-helix after the destruction of R241–E200 bond; the effect in question was demonstrated in a series of simulations [23]. In addition to these conformational changes, event G should also include the formation of interaction between the aromatic rings of the ligand and residue W260 [17, 22] located in the lid region of A-domain. We could not explore this interaction because we used a RI*α*Δ (242–374) mutant.

## 4. Discussion

The transition path we have shown is in good agreement with various experimental or simulational data. Firstly, event G taken in the opposite direction completely matches the straightening of B/C-helix accompanied by R241-E200 disruption which was demonstrated in a series of MD simulations [23]. Secondly, NMR analysis of EPAC interaction with its reverse agonist Rp-cAMPS, partial agonist N6-phenyl-cAMP, and agonist cAMP provided insight into the sequence of stages along the cAMP-binding domain transition path [3]. According to this analysis, tightening of the PBC is prerequisite for N3A-motif and B/C-helix changes that, when combined, can lead to B-conformation (as in the case of cAMP) or remain uncompleted (as in the case of N6-phenyl-cAMP). The last fact occurs due to N6-phenyl-cAMP inability to interact properly with the lid and when being applied to the sequence of events suggested herein may be regarded as a shift of conformational equilibrium toward the state resulting from event F. Thirdly, the confirmation and demonstration of the hinge model proposed by Rehmann et al. supply evidence for the suggested transition path [2, 3]. Events B, D, and E correspond to hinge motion and provide a rational explanation for the mechanism triggering this process.

By contrast, the model that assigns the main part in initiating A-domain conformational transition to electrostatic relay R209-D170-R226 [4, 5] was not confirmed by our simulations. It seems that this relay plays another role pertaining to the stabilization of B-conformation and H-conformation. The result corresponds with a negligible decrease in PKA function when R209 is replaced with isoleucine [6]. It also agrees with the observation that glutamine homologous to D170 in EPAC cAMP-binding domain does not take part in the conformational transitions [24]. Nevertheless, our study fully confirmed the idea of electrostatic interactions' crucial role in mediating the cAMP-induced processes. A minor displacement of A202 amide group resulting from switching between two hydrogen bonds triggers large conformational changes of the whole domain. Based on this result, we introduced new electrostatic relay cAMP(O6)–A202(N-H)–G199(C=O) that transmits the information on ligand binding to the hydrophobic switch L203–Y229, sets hinge region (a.a. 227–232) in motion, and leads thus to the conformational transition of A-domain.

Our results provide an insight into the possible evolution and functioning of other cAMP-binding domains. Visual analysis of available cAMP-binding domain structures lets us make the following hypothesis about existing transition pathways. The simplest domains, domains of prokaryotic transcription factors and A-domains of EPACs, perform only a part of event B (if the ligand is cAMP) or leave the PBC unchanged (if the ligands are other small molecules). In both cases, the packing of L203 side chain into hydrophobic pocket does not occur, and hinge motion is blocked. N3A-motif in these domains is absent or always keeps its position away from the PBC. The only change possible for these domains is the straightforward movement of the lid region toward the aromatic moiety of a ligand and binding to it. All events, except for F and G, can be observed in cAMP-binding domains of ionic channels (CNG or HCN). Instead of these events, one may expect to observe single-stage hinge motion that allows the lid to move closer to the base of a ligand and interact with it. В-domains of EPACs and A-domains of PKAs are the only domains which have the united B/С-helix in H-conformation and so perform events F and G. The difference between these domains lies in the position of N3A-motif, as it was mentioned before. B- and A-domains of PKAs are very much alike, but B-domains have Pro-Gly hinge between B- and C-helices. This hinge undergoes substantial conformational transition when domains pass to B-conformation, so one or several events that account for these unique structural changes should follow after event E. An important characteristic of both domains of PKAs and B-domain of EPACs is the shortness of B-helix (two turns compared with three turns observed for other cAMP-binding domains). It seems that either one group of domains lost one turn of helix or the other group acquired it. Taking into consideration the most complicated pathways we can propose for domains with short B-helix and great variability of ligands common to domains of prokaryotic transcription factors, we hypothesized that cAMP-binding domains of PKAs and B-domain of EPACs are new steps in evolution.

## Supplementary Material

Supplementary material comprises two files. The PDF contains definition of collective variables and specification of thirteen productive trajectories. The Movie file represents animated sequence of transition events shown in Figures 1-4.

## Abbreviations

PKA: Protein kinase A
RI*α*: Regulatory subunit I*α* of protein kinase A
cAMP: 3′-5′-Cyclic adenosine monophosphate
PBC: Phosphate binding cassette
MD: Molecular dynamics
RMSD: Root mean square deviation (distance)
PCA: Principle component analysis.
